# The Use of Cyanoacrylate and Glubran in Dentistry: A Review of Clinical Applications and Outcomes

**DOI:** 10.3390/ma18112642

**Published:** 2025-06-04

**Authors:** Michele Miranda, Francesco Gianfreda, Graziana Molica, Mirko Martelli, Marco Gargari, Patrizio Bollero

**Affiliations:** 1 Department of Clinical Sciences and Translational Medicine, University of Rome “Tor Vergata”, 00133 Rome, Italymirko.marte@libero.it (M.M.); marco.gargari@gmail.com (M.G.); 2 Department of Systems Medicine, University of Rome “Tor Vergata”, 00133 Rome, Italy

**Keywords:** cyanoacrilate, Glubran, oral surgery, oral wound

## Abstract

Cyanoacrylate-based adhesives have gained increasing attention in dentistry for their rapid polymerization, biocompatibility, and antimicrobial activity. This review analyzes the clinical use of cyanoacrylate adhesives—particularly the Glubran II formulation—in dental procedures, including wound closure, tissue management, and bleeding control. A comprehensive literature search was conducted across PubMed, Scopus, and Web of Science databases for studies published between 2000 and 2024, using specific inclusion criteria (clinical and in vitro studies focusing on dental applications of cyanoacrylates) and exclusion criteria (non-dental uses, insufficient data). The findings indicate that compared to traditional sutures, cyanoacrylates, especially n-butyl and octyl derivatives, significantly reduce operative time, postoperative pain, and infection rates. However, differences among formulations—such as degradation rate and cytotoxicity—require further exploration. Glubran II, in particular, shows promising results in hemostasis and wound stability. This review highlights the potential of cyanoacrylate adhesives as effective, minimally invasive alternatives in dental surgery and underlines the need for standardized protocols and long-term comparative studies.

## 1. Introduction

The cyanoacrylate adhesive, first synthesized in 1949 by Ardis, found its pioneering medical application in 1959 when Howard et al. [1] introduced it for surgical purposes. Its importance was further emphasized during the Vietnam War in the 1960s, where it served as a crucial tool for stopping hemorrhages among American soldiers on the battlefield [1]. The medical literature has documented successful applications of cyanoacrylates in various scenarios, such as in the closure of abdominal and gastrointestinal fistulas, oroantral communications, the adhesion of graft materials in regenerative oral surgery, and the repair and hemostasis of parenchymal tissues in hemorrhagic injuries or lacerations of the liver, kidney, and pancreas. These reports highlight their efficacy as durable adhesives in medical procedures [2].

Eastman 910^®^ (methyl-2-cyanoacrylate), the first commercial cyanoacrylate adhesive, was released in 1958. However, its medical use raised issues of toxicity. Subsequently, superior homologues were developed and applied in various surgical procedures, exploiting their ability to polymerize on moist surfaces. Researchers Leonard, Kulkarni, Brandes, Nelson, and Cameron proposed that the cytotoxicity of these materials stems from the release of toxic degradation products and varies with the polymer degradation rate. Specifically, the methyl polymer exhibits higher cytotoxicity because it degrades more rapidly in vivo compared to other cyanoacrylates studied; the toxicity is believed to be caused by degradation products such as formaldehyde and cyanoacetate, which have led to the identification of more biocompatible homologues, such as butyl cyanoacrylate, characterized by its four alkyl groups in the side chain [3].

Regarding pharmacokinetics, cyanoacrylates can be readily absorbed by the skin and mucous membranes due to their biodegradability and are subsequently excreted through faeces and urine. It is essential to note that cyanoacrylate degradation products are not considered carcinogenic. The degradation of alkyl-2-cyanoacrylate occurs through chemical or enzymatic processes. Chemical degradation in aqueous solutions begins with a hydroxyl ion attack, initiating a reverse Knoevenagel reaction. This reaction, producing formaldehyde and cyanoacetate, breaks the carbon–carbon bond. Alkaline solutions and heat accelerate the degradation process. The degradation rate is generally inversely proportional to the length of the side chain; factors such as polymer surface, particle size, polymer molecular weight, and molecular weight distribution all impact the degradation rate [4].

During the 1970s and 1980s, researchers evaluated the adhesive properties of cyanoacrylates for dental applications, focusing on stability, biocompatibility, and toxicity. It was discovered that early cyanoacrylates, such as methyl cyanoacrylate, exhibited excessive toxicity due to the rapid release of formaldehyde during degradation. This led to the development of more biocompatible variants, including butyl cyanoacrylate and octyl cyanoacrylate. Their applications extended to oral and periodontal surgery, facilitating wound healing without the need for sutures and reducing postoperative trauma.

Since the 1990s, cyanoacrylates have been increasingly employed in regenerative dentistry and implantology.

Clinical applications include the closure of surgical and gingival wounds (reducing the risk of infections and accelerating healing); the sealing of oroantral communications (preventing bacterial passage between the oral cavity and maxillary sinuses); the adhesion of grafting materials and membranes in regenerative surgery; and the management of dental trauma, such as mucosal fractures or the fixation of avulsed teeth.

Among the wide range of cyanoacrylate-based hemostatic agents, an innovative product called Glubran 2 stands out. According to European Directive 93/42/EEC, Glubran II is a Class III CE-certified surgical medical device designed for internal and endovascular use. Composed of N-butyl-2-cyanoacrylate and methacryloxysulfolane, it exhibits remarkable hemostatic and adhesive properties, creating an effective antiseptic barrier upon solidification. It rapidly polymerizes when in contact with living tissue in a moist environment, forming a thin, high-strength elastic coating that promotes strong adhesion [5]. When applied correctly, the material begins to solidify within 1–2 s and completes the solidification reaction within approximately 60–90 s. Glubran II achieves its maximum mechanical strength at the end of this reaction. From the perspective of degradability and cytotoxicity, comparing different cyanoacrylate-based medical devices (N-butyl cyanoacrylate [n-BCA] and octyl cyanoacrylate [OCA]) with the chemical formulation of Glubran II, the latter exhibits an advanced composition based on n-butyl cyanoacrylate modified with acrylic esters. This distinct formulation enhances the polymer’s flexibility while reducing its fragility, enabling controlled polymerization (thereby minimizing the risk of thermal damage), providing excellent hydrolytic resistance, and ensuring optimized adhesion to biological tissues. This product has been utilized for several years across various fields, including neurosurgery, otorhinolaryngology, dentistry, maxillofacial surgery, pediatric cardiac surgery, vascular surgery, general surgery, and thoracic surgery. It can also be applied as a spray in laparoscopic and thoracoscopic surgical procedures [6].

The current article aims to provide a review of the existing literature on the evolution of cyanoacrylate usage, offering a valuable contribution in favour of the use of a specific cyanoacrylate, Glubran II. Specifically, this review seeks to answer the following guiding question: What are the current applications, benefits, and limitations of Glubran II in dental and surgical contexts, and how does it compare to other cyanoacrylate-based adhesives in terms of clinical performance and safety? In addition to the literature overview, the article presents a focused clinical study involving nine patients affected by systemic diseases at the Tor Vergata Hospital in Rome. The study evaluates the effectiveness of Glubran II as an adhesive material for both internal and external applications, highlighting its specific indications in hemostasis control, bone stabilization in regenerative techniques, and the impressive reduction in intraoperative time, which could significantly streamline surgical procedures and improve patient outcomes.

## 2. Materials and Methods

Bhaskar, Jacoway, Margetis, Leonard, and Pani (1966) [7] were among the first to investigate the effects of cyanoacrylate products in oral surgery, testing methyl, ethyl, propyl, and butyl cyanoacrylates on tongue lesions. They found that all cyanoacrylate sprays produced durable hemostasis, but methyl cyanoacrylates were often associated with abscesses and suppuration. Butyl and propyl cyanoacrylates were locally phagocytosed, resulting in relatively low levels of tissue necrosis and abscess formation and rapid and long-lasting hemostasis (Table 1). Subsequent studies have confirmed that longer alkyl chain cyanoacrylates, such as butyl and isobutyl formulations, are more biocompatible due to their slower degradation rates and lower production of cytotoxic byproducts such as formaldehyde and cyanoacetate. In particular, butyl cyanoacrylate has demonstrated superior integration with tissue, minimal inflammatory response, and enhanced healing profiles compared to methyl or ethyl derivatives. The slower biodegradation of butyl-based cyanoacrylates reduces the local accumulation of toxic metabolites, contributing to reduced tissue irritation and a more favourable healing environment. These findings underscore the importance of chain length in determining adhesive performance, biocompatibility, and wound healing dynamics.

In a 1969 study conducted by Ochstein [8] on cyanoacrylates and other periodontal dressings for gingival surgical wound healing in Beagle dogs, it was found that butyl cyanoacrylate demonstrated more satisfactory healing in clinical evaluations, particularly in animals treated with split-flap techniques. In 1967, King, Reynolds, and Kruger [9] studied the hemostatic effect of methyl cyanoacrylate following tooth extractions in both heparinized and non-heparinized dogs. They discovered that long-term hemostasis could be achieved using acrylic plugs coated with a thin adhesive layer.

Bhaskar, Frisch, Cutright, and Margetis (1967) [10] used adult rats to study the impact of butyl cyanoacrylate on the healing of extraction wounds. Half of the wounds were covered with a butyl cyanoacrylate spray, while the others were left uncovered. Compared to control wounds, the inflammatory infiltrate was consistently lower in wounds protected with cyanoacrylate spray.

In 1971, Jandinski and Sonis [11] conducted an in vitro study on the effects of isobutyl cyanoacrylate on four types of bacteria, observing bacterial growth inhibition in *Streptococcus*, *N. catarrhalis*, *Gaffkya*, and *S. aureus* when the polymer was applied to Petri dishes. Active colony reduction was noted only with Streptococcus following polymer application. The relative cytotoxic effects of methyl, isobutyl, and octyl cyanoacrylates on L929 fibroblasts were compared by De Renzis and Aleo (1970) [12]. The study revealed that the zones of cytotoxicity were most extensive around methyl cyanoacrylate-saturated disks, while those surrounding the other cyanoacrylates were relatively smaller. After 6–12 h of exposure, isobutyl and octyl cyanoacrylates produced no visible reactions. Still, after 12, 24, and 36 h, toxic areas around the methyl disks expanded rapidly, while those around isobutyl and octyl disks continued to grow but did not reach the size of the methyl zones. The study concluded that all tested cyanoacrylates exhibited some toxicity, with methyl cyanoacrylate causing the most severe cell death compared to isobutyl cyanoacrylate [12].

These early observations have been confirmed by more recent studies, which consistently report lower cytotoxicity levels associated with butyl and isobutyl cyanoacrylates. For instance, Kondoh et al. (2003) [13] and Vastani et al. (2013) [14] corroborated the biocompatibility and reduced inflammatory response of these derivatives, reinforcing the notion that the type of cyanoacrylate used significantly influences tissue response.

Research continued in subsequent years with studies by Mehta et al. [15], who used butyl cyanoacrylate for osteosynthesis in ten cases of mandibular fractures, with follow-up periods of 1 and 6 months, reporting no adverse reactions or chromosomal alterations.

Perez’s study [6] in 2000 was fundamental, documenting the use of cyanoacrylates in over 100 cases of dental extractions or apical/periodontal surgeries without adverse reactions. In 2003, Kondoh et al. [13] applied cyanoacrylate to hemostatic gauzes on alveolopalatal wounds following alveolar bone grafts, reporting the absence of significant signs of inflammation or wound dehiscence in treated sites. In 2013, Vastani et al. [14] replicated the experiment using syringes for cyanoacrylate application in palatoplasty procedures, confirming previous results.

In 2006, Cooper and Paige [16] studied the effectiveness of cyanoacrylates in cleft lip and palate surgeries in children and adults. Later, in 2014, Salata et al. [17], in a short-term study, used n-butyl cyanoacrylate to fix autologous grafts in the mandibles of rabbits, comparing the results with screw fixation over 4–8 days. The evaluation revealed less inflammation and greater mineralized tissue volume in cases treated with cyanoacrylate. However, in 2017, a study published in the *Journal of Oral Implantology* by De Santis et al. [18] on the use of cyanoacrylate to secure a block bone graft to the mandible involved 24 rabbits over a 40-day evaluation period. Results showed that biological integration with the bone tissue was not achieved while the graft remained stable (Table 2).

A pioneering study in periodontology was conducted by Giray et al. in 1997 [4], where cyanoacrylate adhesives were used in root sections. Rezende et al. [19] later used cyanoacrylate to secure a resorbable membrane for guided tissue regeneration, observing favourable aesthetic results four years after the procedure. Subsequently, Kulkarni et al. [20] published a study on cyanoacrylate adhesives in 24 patients undergoing flap procedures for pocket therapy, reporting faster healing in sites treated with adhesive compared to sutured sites, particularly in the first week. After 21 days to 6 weeks, the healing in both sites was almost identical. Further studies in periodontology included those by Agilli [21], who used cyanoacrylate adhesives in free gingival grafts, and by Ranson (2016) [22], who applied cyanoacrylate to stabilize pedicle flaps. In 2017, Ozcan [23] reported the effectiveness of cyanoacrylate in improving palatal wound healing after free gingival grafts.

In surgical research, H. Choi et al. [24] explored the effectiveness of cyanoacrylate adhesives for repairing sinus membrane damage. Their study involved six rabbits, where one side of the maxillary sinus was treated with cyanoacrylate, and the opposite side was left untreated. Two weeks later, the evaluation showed that the treated side had developed a new epithelial layer over the perforation, while the untreated side displayed signs of sinusitis. In 2013, Y. Sagara [25] reported on the use of cyanoacrylates for selective transarterial embolization to manage bleeding after third molar extractions. Further evidence supporting the use of cyanoacrylates came from Bozkurt and Saydam’s 2008 [26] study, which assessed their application in head and neck surgeries. This study involved 80 patients and demonstrated positive outcomes, with no postoperative complications and high patient satisfaction regarding scar appearance.

Although the literature is still limited, substantial scientific evidence supports the use of Glubran II in various medical fields. According to the study by Kull et al. [27], which aimed to investigate its in vitro characteristics in comparison with a widely used fibrin-based adhesive (Tissucol, Baxter Healthcare, Deerfield, IL), polymerized Glubran II appeared as a consistent, uniform, slightly granular, opalescent film. In contrast, Tissucol appeared as a gelatinous, viscoelastic material that was difficult to distribute evenly on the skin and had poor adhesion. The different antimicrobial action between conventional fibrin glues and Glubran II stems from their distinct chemical formulations. As the studies above indicate, isobutyl cyanoacrylate exhibits significant antimicrobial activity, particularly against pathogens such as *Staphylococcus aureus* and *Escherichia coli*. Glubran II, enriched with modified ester groups, facilitates rapid polymerization, creating a solid barrier that effectively prevents bacterial penetration. The chemical composition of Glubran II is characterized by N-butyl-2-cyanoacrylate and metacryloxysulpholane (MS), a monomer specifically added to improve flexibility and reduce cytotoxicity. The presence of MS enhances the adhesive’s polymerization kinetics and modifies its degradation profile, resulting in a more controlled and biocompatible behaviour upon application to biological tissues. The formulation of Glubran II is designed to initiate polymerization upon contact with humid environments, such as living tissue, where the glue transforms from liquid to solid in seconds, forming a flexible, transparent film. This unique property of Glubran II could potentially revolutionize infection control in surgical procedures. In contrast, fibrin glue-based medical devices lack these properties; they do not directly prevent microbial colonization and may provide a substrate for colonization, as they fail to form a rigid barrier over the wound. In the study by Ersoy et al. [28] involving 40 rats, cyanoacrylate significantly reduced local bleeding and overall surgery time compared to the traditional suture-based closure of an arteriotomy. Buric has also used Glubran II as a tissue adhesive to close oroantral communications. The study, conducted on healthy patients treated with closure techniques using both sutures and Glubran II tissue adhesive, showed the complete epithelialization of the wounds, whether sutured or treated with Glubran II, after 23–25 days. However, the study observed that in an open biological environment such as the oral cavity, Glubran II required a longer time to be eliminated from the application site than its use in internal sites. The study aimed to evaluate two main concerns regarding Glubran II: its impact on alveolar bone tissue and its biological fate. Thus far, experimental results indicate a lower risk of damage than other adhesives such as Histoacryl, particularly in studies involving the embolization of arteriovenous fistulas and the resection of lung tissue in animal models. The aerostatic effects of Glubran II on lung tissue are biologically advantageous and comparable to traditional mechanical suture techniques, suggesting its safety for clinical applications (17). Regarding its effect on bone tissue, Glubran II was used to stabilize bone grafts in the repair of defects in the anterior cranial fossa in a case involving a 12-year-old boy with a brain injury. The subsequent development of a brain abscess caused by *Streptococcus pyogenes* was successfully managed without complications following the application of Glubran II on the bone tissue near the severe infection sites [28].

A significant study published by the Cairo Faculty of Medicine, Egypt, investigated the use of Glubran II in uvulopalatoplasty procedures. The study aimed to assess the stability of the soft palate tissue following adhesive application and postoperative pain levels compared to sutures and to achieve a less invasive procedure, thereby reducing morbidity during soft palate healing and improving surgical outcomes. The study, conducted on 54 patients divided into two groups, showed that postoperative pain was significantly lower in the adhesive group compared to the suture group during the two-week post-surgery period. After a six-month follow-up, no changes in the configuration of the palate were observed. The study emphasized the adhesive nature of the glue, which played a crucial role in maintaining the stability of the soft palate flaps throughout the healing period, preventing flap displacement, and ensuring optimal outcomes for the newly formed soft palate margin. In contrast, sutures in various palatoplasty techniques presented several disadvantages, including prolonged intraoperative time, difficulty accessing challenging areas, increased postoperative pain, and a higher incidence of palatal fibrosis. Additionally, the hemostatic properties of the material reduced bleeding between the soft palate flaps, decreasing the risk of postoperative infections [29].

Further scientific evidence was provided in 2010 with the work of Piero Balleri [30], which aimed to describe the survival rate of Astra Tech implants inserted using the sinus membrane elevation technique without biomaterials in a series of 15 patients with atrophic posterior maxillae, followed clinically and radiographically for one year after loading. In this study, Glubran II was used to stabilize the bone window at the end of the procedure. The study concluded that the technique used for implant rehabilitation was successful one year after the intervention. Glubran II effectively and rapidly stabilized the bone window, ensuring immobility during the flap repositioning and suturing. This method was particularly useful in poorly angled osteotomies with rounded margins that could complicate the correct repositioning of the bone window. No advantages were noted from a strictly regenerative standpoint: the technique did not use biomaterials. Still, it relied on the formation of a blood clot, which served as a scaffold and provided cellular elements and substances to promote bone regeneration. Using a hemostatic agent like Glubran II ensured the formation of a highly stable blood clot, playing an important role in subsequent bone regeneration processes [30].

The use of cyanoacrylate-based tissue adhesives such as Glubran II requires the assessment of these materials’ mechanical behaviour and long-term stability when in contact with human tissues. Research on these aspects has evolved, beginning with Paéz’s 2005 [31] study published in the *Journal of Biomaterials Applications*. This study aimed to determine whether Glubran II, alone or in combination with sutures, could provide sufficient mechanical strength and long-term stability for use on inert tissues, such as bioprostheses or implants, in cardiovascular surgery. The tests were designed to determine the tensile strength of the calf pericardium samples, a material similar to that used in the manufacturing of bioprosthetic heart valve leaflets. The samples were cut, bonded with overlapping tissue, or sutured, and reinforced at the suture holes with the same adhesive. One hundred thirty-two tests were conducted on three samples: intact tissue (control), samples cut and bonded with Glubran II, and samples cut and sutured with reinforcement at the suture holes with Glubran II. Seven days after the procedure, 12 samples from each group, including controls, underwent tensile testing to failure, and the results were compared. In the stability study, both the Glubran II-only group and the suture-and-adhesive group were subjected to tensile tests up to failure at 30, 60, 90, and 120 days. The results showed that bonding with the adhesive provided a tensile strength ranging from 1.04 to 1.87 kg, likely insufficient for use in heart valve leaflets but offering a high degree of elasticity. After 120 days, both the adhesive-only and suture-plus-adhesive groups demonstrated excellent elastic behaviour, with no stiffness or hardening of the pericardium. Tensile strength and breaking load tests showed excellent performance in the sutured and adhesive-reinforced samples, with stability maintained after 120 days. In contrast, the adhesive-only samples exhibited a significant loss of strength. No loss of elasticity due to secondary hardening of the adhesive was observed, which would be undesirable for the production of heart valve leaflets. Therefore, in evaluating Glubran II as a valid tissue adhesive in cardiovascular surgery, the study concluded that the adhesive provided significant bonding strength, although likely too low for effective heart valve bonding, while maintaining a high degree of elasticity in tissue samples subjected to tensile tests. Furthermore, the application of an adhesive at suture holes in calf pericardium improved its resistance to tearing, maintaining excellent elasticity and possibly contributing to the anisotropic behaviour of the tissue [31].

The study by Paéz [31] inspired numerous subsequent publications, further supporting cyanoacrylate-based adhesives in surgical settings. In 2009, a confirmation of Paéz’s conclusions was published in the *Journal of Surgical Research* by the Institute of Clinical Physiology—CNR (Massa, Italy)—which conducted adhesive and mechanical tests on Glubran II to characterize its behaviour, providing useful insights for its application. The study compared Glubran II with a widely used commercial fibrin glue (Tissucol, Baxter Healthcare, Deerfield, IL, USA). Both products were applied to biological tissues to test their adhesive properties, and after complete polymerization, samples underwent various load tests simulating in vivo conditions [32]. Pig skin was used as the standard substrate, and each test was repeated three times. The results showed that the polymerized Glubran II films resisted peeling from the skin strips, remaining intact under maximum load, thus ensuring uniform stress distribution across all bonded surfaces. In contrast, Tissucol samples showed weak bonding, with peeling occurring early in the test, and the fibrin clot broke at low tension values, concentrating the stress at anchor points [33,34]. Specifically, the Glubran II films hindered the separation of materials under stress, with breakage occurring due to the detachment of the Glubran II films from the tissues rather than the fragmentation of the films themselves. This led to the conclusion that the adhesive bonds were stronger than those established with the skin, while the Tissucol samples exhibited no significant resistance to skin detachment at any stage of the test [35] (Table 3).

The study concluded that Glubran II demonstrated excellent tissue penetration and high adhesive capacity, though somewhat lower than the intrinsic adhesion of the material itself. In almost all cases, the integrity of the samples was maintained until rupture, and the elasticity of the samples was preserved [36]. Furthermore, maintaining tissue elasticity after Glubran II bonding reduced the internal stress commonly generated by sutures, which often leads to the limited durability of certain implants and procedures. The study reinforced previous findings, demonstrating the integrity of cyanoacrylate-based tissue adhesives, particularly Glubran II, and recommending their utility in surgical and endoscopic practice, highlighting their excellent hemostatic action, strong tensile resistance, and capability to prevent partial tissue loss [37] (Table 4 and Table 5).

## 3. Results

Further evidence supporting the existing literature on Glubran II is provided by a study conducted at the Department of Special Odontostomatological Pathology of the Tor Vergata University Hospital in Rome, Italy under the direction of Professor Patrizio Bollero. The study, carried out by M. Miranda’s team, aimed to assess the efficacy of Glubran II as a hemostatic agent and its stability as a tissue adhesive in oral surgery procedures in patients with cardiovascular diseases. The objectives of the study included testing the material’s stability, evaluating the potential to reduce intraoperative time and postoperative pain, assessing the use of Glubran II in stabilizing graft material in regenerative techniques, and evaluating its effectiveness as a first-line hemostatic agent in a hospital setting.

**Figure 1:**
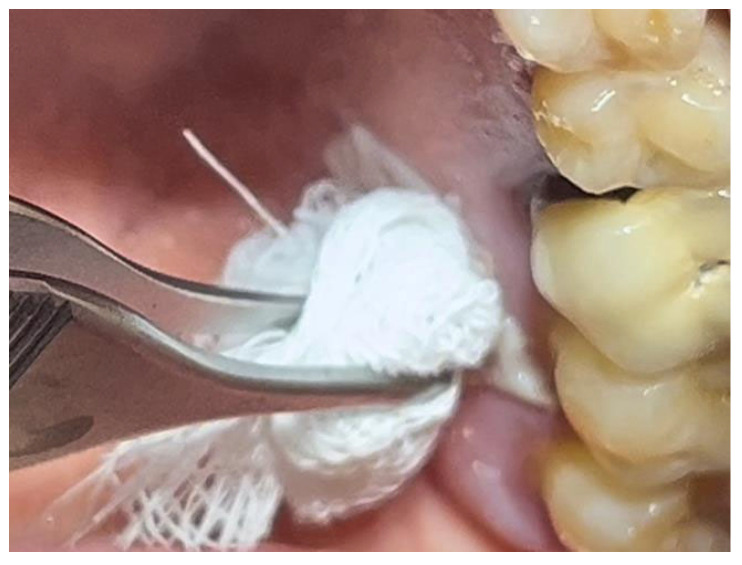
Lesion resembling leukoplakia localized on the palate, non-removable through biopsy by scraping.

**Figure 2:**
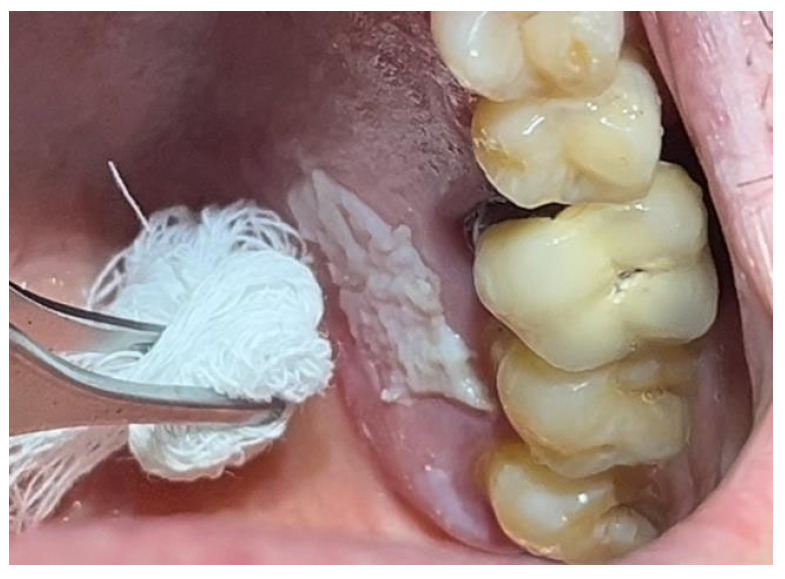
Leukoplakia-like lesion localized on the palate following scraping with a sterile gauze.

The study involved nine patients diagnosed with cardiovascular conditions, selected according to clearly defined inclusion criteria: presence of diagnosed cardiac conditions, the need for oral surgery, and signed informed consent. Exclusion criteria included patients with known allergies to the components of Glubran II, active infections, or systemic conditions contraindicating surgery. All patients were administered an antibiotic regimen consisting of Amoxicillin + Clavulanic Acid (875/125 mg), GlaxoSmithKlin, London, UK starting 24 h prior to the procedure, which was performed after obtaining cardiology clearance. Excellent results in terms of managing intra- and postoperative bleeding and tissue healing were observed in a 67-year-old patient with hypertension and diabetes mellitus, who underwent an excisional biopsy of a white lesion located on the palate with a leukoplakic appearance. The lesion, located on the palatal vault, appeared white, non-glossy, with undefined margins and soft consistency, and was non-removable via scraping biopsy (Figure 1 and Figure 2). A decision was made to surgically remove the lesion with wide incision margins to provide a biopsy sample (containing the lesion with surrounding healthy tissue) to the Department of Pathological Anatomy at the University Hospital of Rome Tor Vergata.

**Figure 3:**
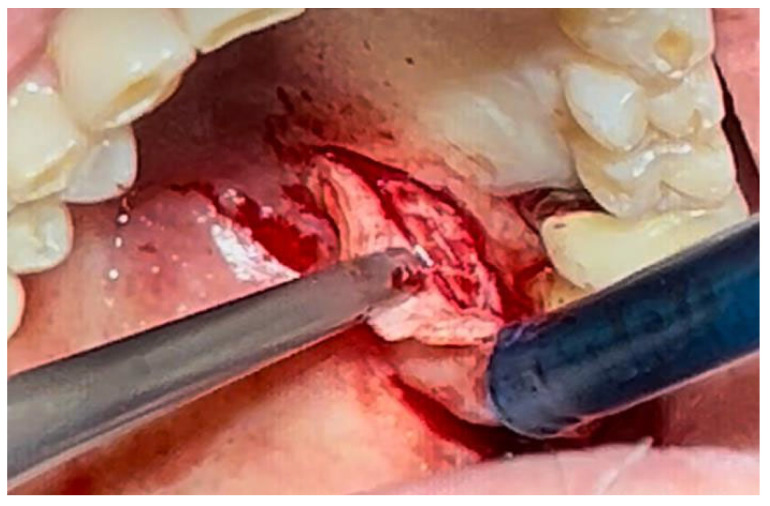
Surgical excision of the white lesion located on the palate with wide safety margins.

**Figure 4:**
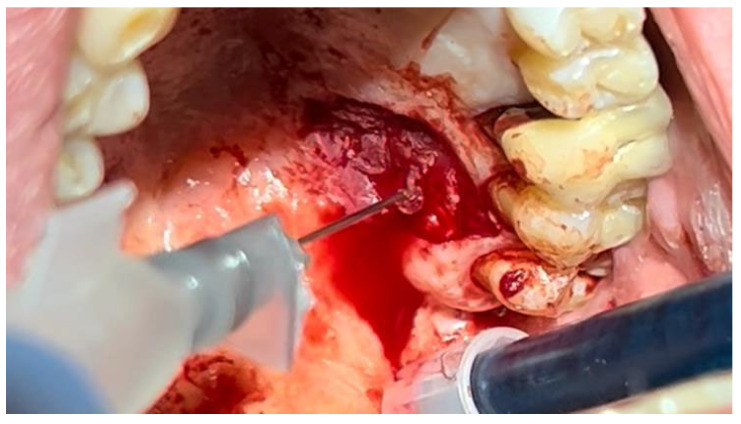
Hemostasis control at the surgical site following excision of the lesion with Glubran II using an endodontic needle.

**Figure 5:**
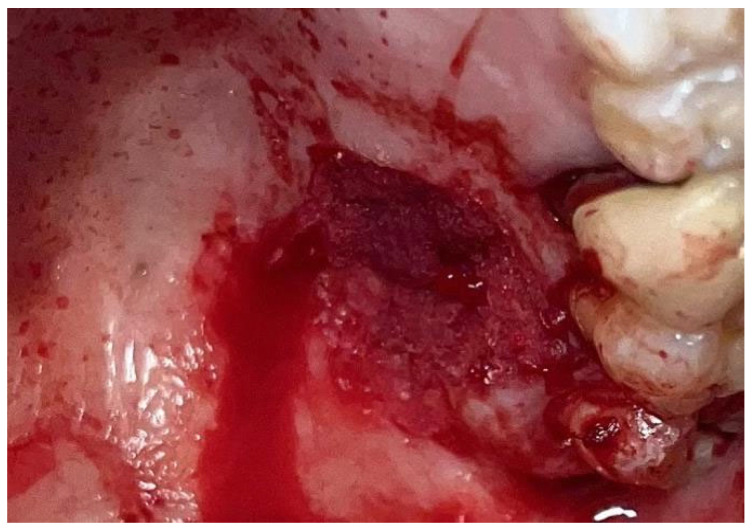
Surgical site following application of Glubran II.

The patient was prepared for the surgical procedure with antibiotic therapy (Amoxicillin + Clavulanic Acid 875/125 mg) and 2% Chlorhexidine rinses starting the day before the surgery. Following the guidelines for patients with hypertension (Figure 1), the excision was performed with the complete removal of the lesion, adhering to standard surgical procedure techniques (Figure 3). Post-excision, hemostasis was achieved using Glubran II surgical glue. In this case, an endodontic needle was used for material application, allowing single droplets to be applied directly to the surgical site (Figure 4). Hemostasis was rapidly achieved in the areas where the glue was applied. The material was carefully deposited around the perimeter of the surgical site, ensuring individual droplets were placed without excessive application (Figure 5).

**Figure 6:**
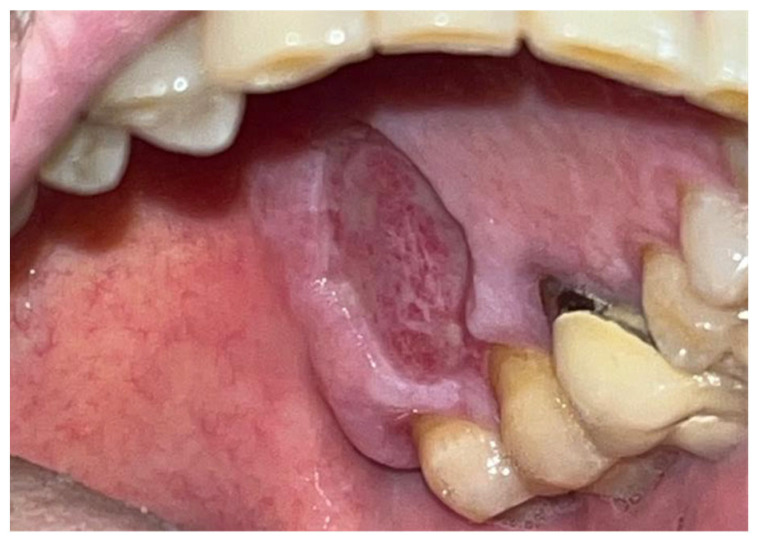
Surgical site day 15 post-surgery.

This technique resulted in optimal hemostasis within seconds, creating a stable, chemically and mechanically secure film protecting the surgical site during the healing period. The patient was re-evaluated 2 weeks post-surgery and reported no discomfort from the glue application, describing the film as hard but not interfering with any essential functions. Over the following two weeks, the film progressively degraded until it was eliminated. The degradation of the film allowed for the formation of new, mature epithelial tissue at the surgical site, with no signs of inflammation or suppuration in the surrounding tissues, though traces of Glubran II remained within the lesion site (Figure 6).

**Figure 7:**
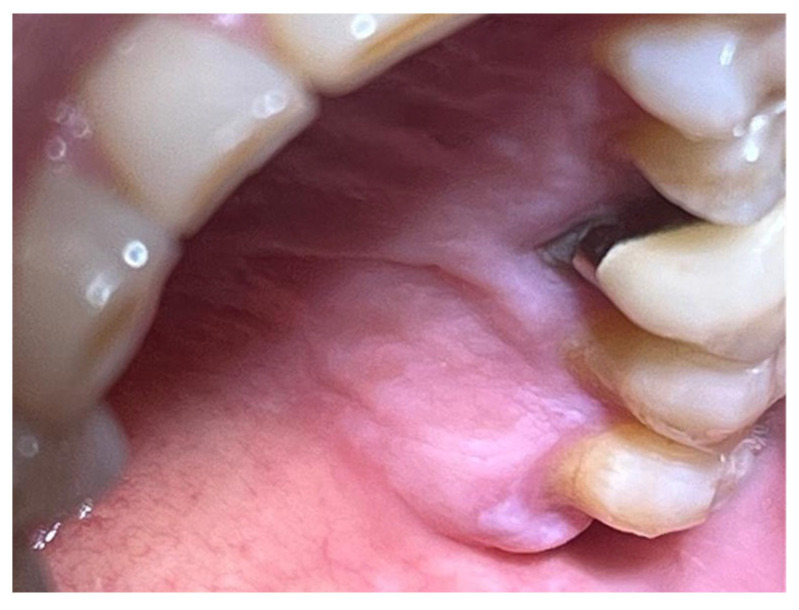
Surgical site day 30 post-surgery.

One month post-surgery, the wound was fully healed, with new keratinized epithelial tissue in the final stages of maturation within the wound site. The newly formed tissue appeared clinically normal, with a uniform pink colour, and no signs of inflammation or infection complicating the healing process (Figure 7).

Thus, Glubran II proved to be a highly effective aid in this elective procedure. It was useful in controlling intraoperative bleeding, reducing the overall surgical time (by eliminating the need for manual hemostasis techniques), and in managing postoperative bleeding, which reduced the risk of complications and provided reassurance to the patient, especially given their hypertension. Furthermore, it formed a protective film over the healing site during the most delicate early stages of tissue regeneration, minimizing the risk of bacterial infections.

**Figure 8:**
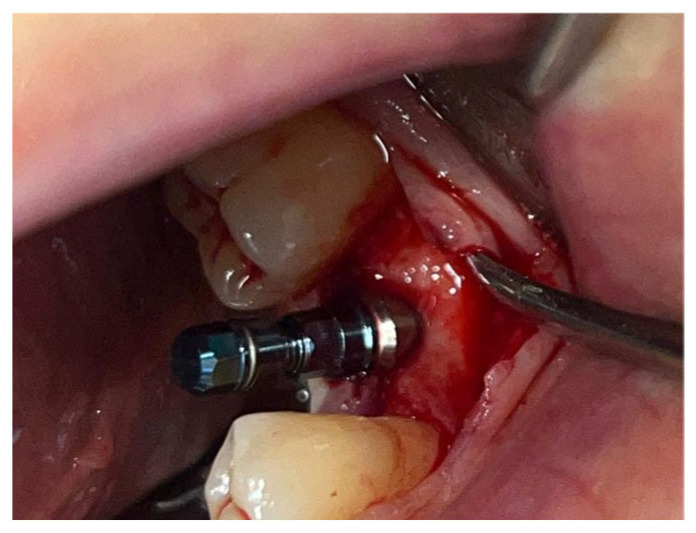
Implant-prosthetic rehabilitation at site 36; buccal bone level prior to regenerative techniques.

**Figure 9:**
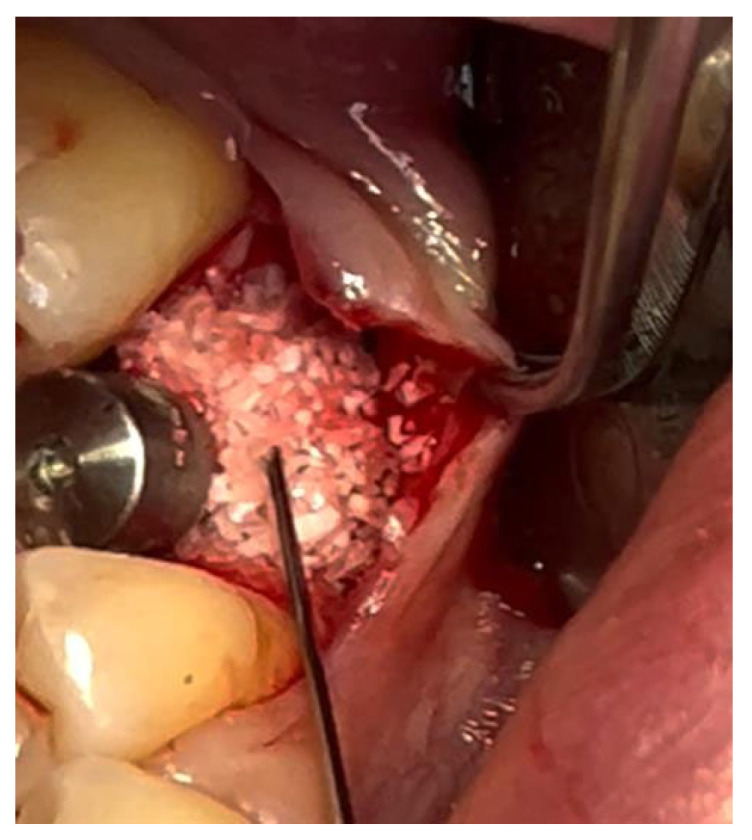
Stabilization of bone regeneration material with Glubran II.

**Figure 10:**
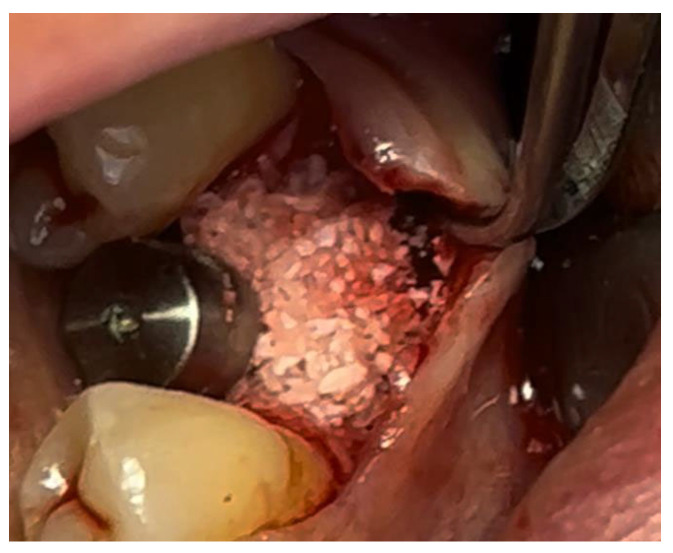
Surgical site at the end of the polymerization time of Glubran II; the site was subjected to tensile testing, with positive results under the applied tensions.

In a 49-year-old patient with ischemic heart disease, Glubran II was used as a stabilizing material for natural bone graft granules at a site rehabilitated with implant-prosthetic treatment, with the aim of achieving volumetric enhancement of the buccal bone. Following specific guidelines for surgical treatment of ischemic heart disease (4), implant therapy was carried out (Figure 8). Natural bone graft material (Bio-Oss^®^—Geistlich, Baden-Baden, Germany) was applied and stabilized with Glubran II. The application was made using an endodontic needle, depositing single droplets of the glue to cover the entire graft material before placing the prepared flap on top (Figure 9). This technique achieved stable graft fixation at the recipient site, demonstrating the efficacy of Glubran II for internal use (Figure 10).

During the postoperative period, no complications were reported, and no signs of material rejection or inflammation at the surgical site occurred. The site was sutured with absorbable sutures (Ethicon Vicryl Rapid 4/0, Johnson & Johnson, New Brunswick, NJ, USA), and soft tissue healing was completed within 15–20 days. A proper evaluation of graft integration and potential bone regeneration will require 3–4 months, but the current results show Glubran II’s effectiveness in stabilizing the graft and preventing inflammation, both in the bone and soft tissue, making it a valuable tool for socket preservation techniques.

**Figure 11:**
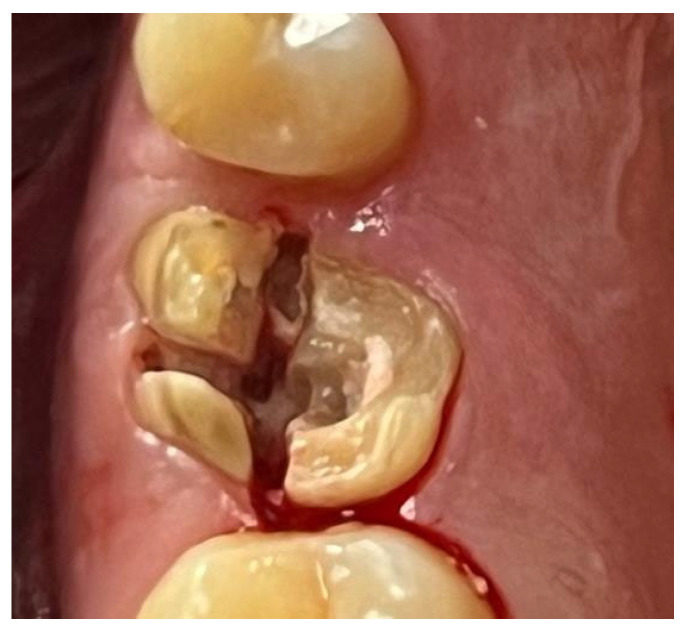
Surgical extraction of 1.6.

**Figure 12:**
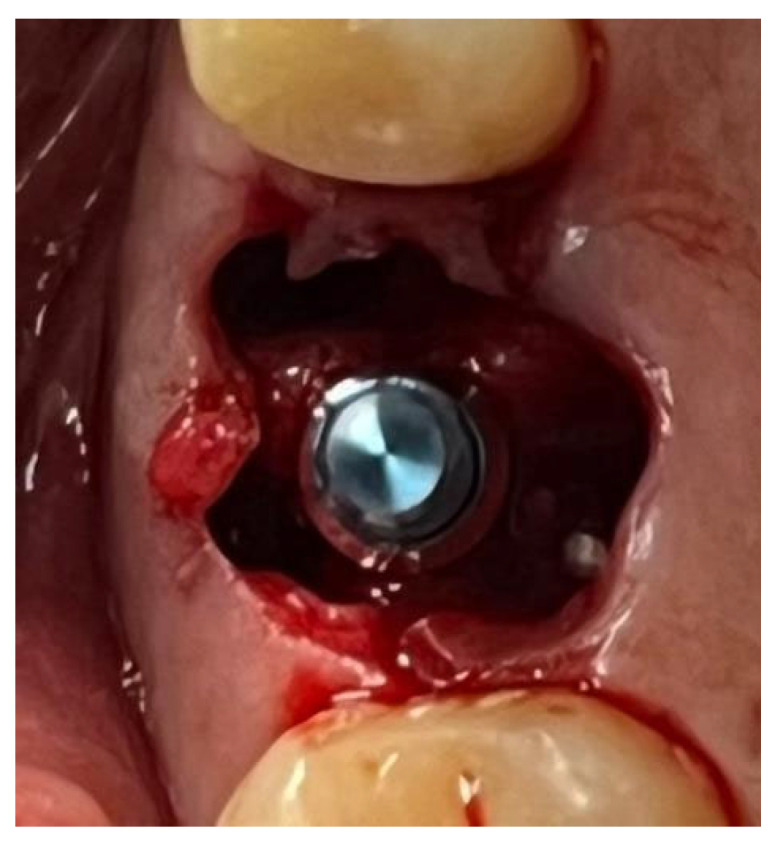
Implant-prosthetic rehabilitation at the surgical site 1.6.

**Figure 13:**
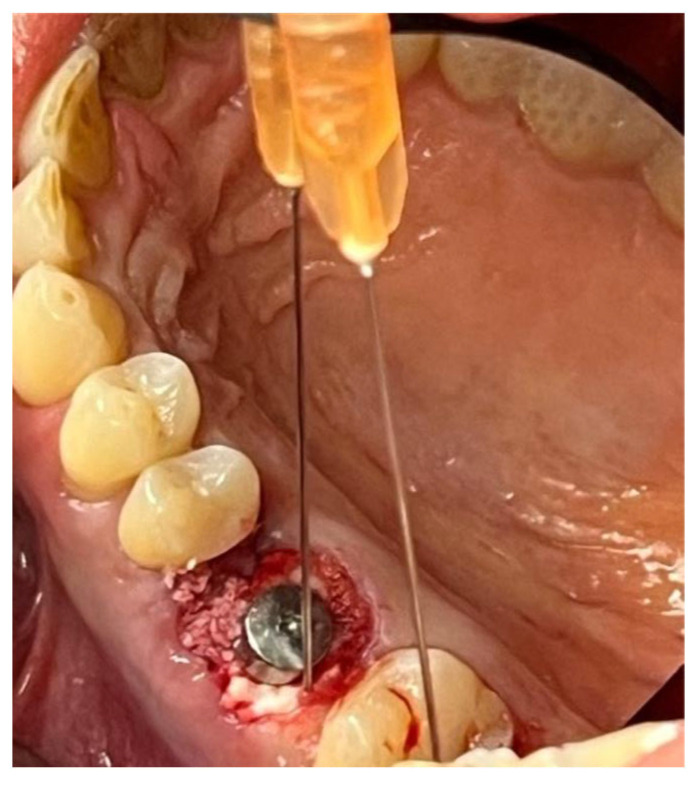
Stabilization of synthetic bone material at the surgical site, undergoing implant-prosthetic rehabilitation with Glubran II using an endodontic needle.

**Figure 14:**
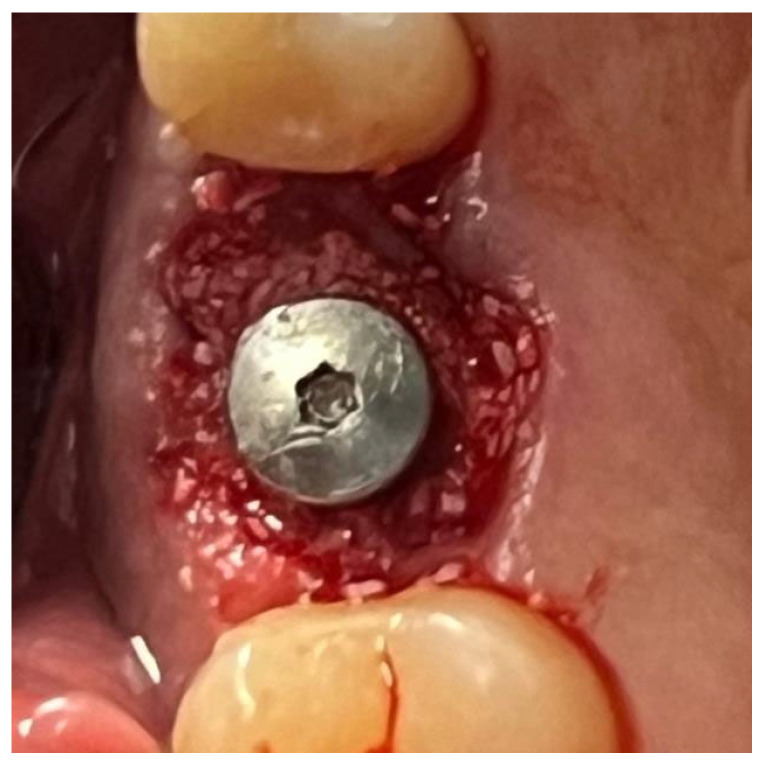
Surgical site after the complete polymerization of Glubran II.

A similar technique was used in a 45-year-old patient with controlled hypertension, treated for the extraction of tooth 1.6 (Figure 11) and subsequent post-extraction implant-prosthetic rehabilitation (Figure 12). The surgical site was treated with bone graft material (Bio-Oss^®^—Geistlich), and a small amount of Glubran II was applied (Figure 13). Once again, Glubran II proved to be highly effective in stabilizing the graft material at the surgical site, providing excellent hemostasis and strong mechanical resistance from the film formed after polymerization (Figure 14).

**Figure 15:**
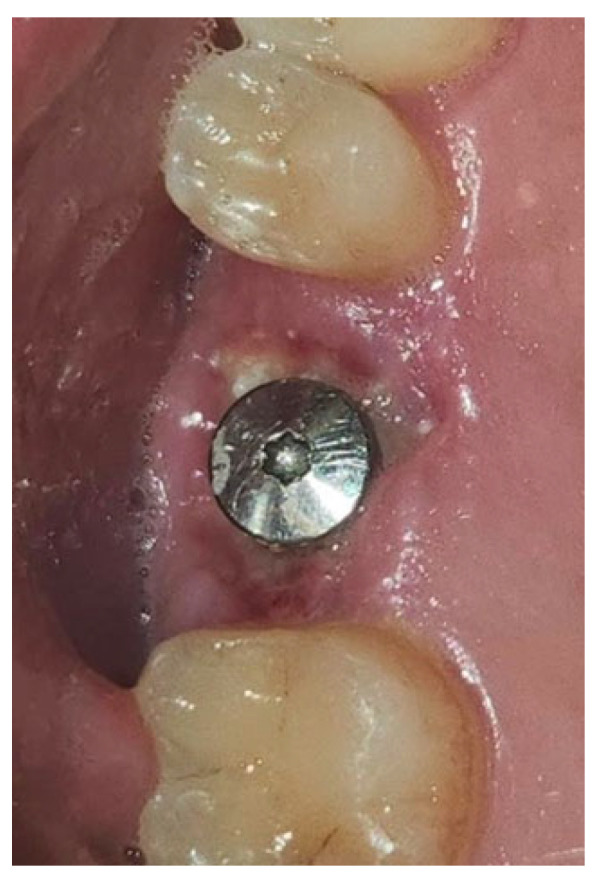
Surgical site one week after surgery.

One week after surgery, no signs of inflammation or suppuration were observed at the site. New tissue formation was noted within the rehabilitated site (Figure 15). The patient reported no discomfort related to Glubran II use, and bleeding was controlled during the surgery, thus reducing the risks associated with the patient’s hypertensive condition.

The study conducted at the University of Rome Tor Vergata highlights several advantages over traditional hemostatic techniques: rapid action and the ability to create a stable and durable physical barrier against hemorrhages (Table 6). It also demonstrated positive results as an adjunct for stabilizing graft materials and resorbable membranes in bone regeneration techniques. The product proved effective as a sealant for post-extraction sockets and, due to its excellent adhesive properties, enabled the approximation of surgical flaps without the need for sutures, significantly simplifying the operative process and improving healing times. The reduction in procedure time is particularly critical for cardiac patients, for whom controlling anxiety and minimizing the use of anesthetics with vasoconstrictors is of paramount importance.

## 4. Discussion

Cyanoacrylate-based adhesives, particularly Glubran II, have emerged as transformative tools in dentistry and broader surgical applications. Their utility stems from their rapid polymerization, biocompatibility, and unique ability to create a protective barrier while facilitating hemostasis. Key studies, such as those by Bhaskar et al. (1966) [7,10], have demonstrated their effectiveness in oral surgery, where butyl cyanoacrylate outperformed earlier formulations by reducing complications like abscess formation and promoting stable hemostasis. These early findings laid the groundwork for their adoption in periodontal, maxillofacial, and other dental applications [38,39].

Glubran II has demonstrated significant benefits over traditional suturing techniques in periodontal procedures. The work of Kulkarni et al. [20] highlighted its role in reducing inflammation and enhancing wound stability during the critical early phases of healing. This aligns with Ranson’s observations (2016) [22], who emphasized its hemostatic properties and ability to stabilize surgical flaps, particularly in challenging clinical scenarios like free gingival grafts. Importantly, these studies underscore how cyanoacrylate adhesives improve patient comfort by minimizing postoperative pain and complications.

Recent clinical evaluations also report that patients treated with cyanoacrylate adhesives experience shorter healing times, less postoperative swelling, and improved comfort compared to traditional sutures, particularly in procedures involving mucosal incisions or graft stabilization (Howard et al., 1973) [1]. These outcomes make them especially valuable for patients with dental anxiety or low pain tolerance.

From a broader perspective, the antimicrobial properties of cyanoacrylates, as investigated by Jandinski and Sonis [11], add a crucial layer of protection to oral surgical environments prone to infection. These properties enhance healing outcomes and reduce the risk of secondary infections, which can complicate recovery in immune-compromised patients or those with underlying systemic conditions.

Even so, although cyanoacrylate adhesives possess inherent antimicrobial activity, especially in the case of Glubran II due to its rapid polymerization and dense sealing effect, some studies have raised concerns about their potential to serve as substrates for biofilm formation in persistent wound environments. While current evidence does not suggest a high risk of bacterial resistance, long-term exposure to subtherapeutic concentrations—especially in contaminated or poorly vascularized tissues—could theoretically promote biofilm development. Further research is needed to understand this risk better and to identify whether prophylactic strategies or formulation adjustments may be required.

However, limitations persist, particularly in bone-related applications. The study by De Santis et al. (2017) [18] highlighted the adhesive’s inability to achieve biological integration with grafted bone tissue over extended periods. While Glubran II offers initial mechanical stability, its limited role in osteogenesis may restrict its application in more complex reconstructive procedures. This aligns with the findings of Salata et al. (2014) [17], who observed reduced inflammation and greater mineralized tissue volume in short-term evaluations but noted the need for alternative strategies to achieve long-term stability.

While cyanoacrylate adhesives have shown promising results in soft tissue healing, they may not be universally suitable for all oral surgeries. For instance, traditional sutures may still offer more predictable outcomes in procedures requiring deep tissue approximation, prolonged tensile strength, or multi-layer closure. Additionally, in the oral cavity’s high-mobility areas, the adhesive’s mechanical resilience may be challenged. Thus, while they represent a powerful adjunct or alternative to sutures in many scenarios, their use should be tailored to the specific demands of each surgical intervention.

Emerging innovations, such as combining cyanoacrylate adhesives with guided tissue regeneration techniques, as demonstrated by Rezende et al. [19], showcase their potential for broader applications. Additionally, their role in socket preservation and implant stabilization, as reported by Choi et al. [24] and others, represents promising avenues for research. While these applications highlight versatility, the challenge lies in tailoring the formulation and application methods to optimize outcomes for specific clinical scenarios [40,41].

Overall, the scientific evidence affirms the effectiveness of cyanoacrylate-based adhesives in enhancing procedural efficiency and patient outcomes in dentistry. Their ability to reduce surgical time, enhance healing, and minimize complications makes them indispensable in many contexts. However, continued innovation and long-term clinical studies must address their limitations and unlock their full potential.

Environmental factors such as temperature and pH also influence the polymerization kinetics of cyanoacrylate adhesives. Polymerization is known to be exothermic and accelerates with increased temperature, which can enhance adhesion speed but may increase the risk of local thermal damage in sensitive tissues. Moreover, acidic environments have been shown to slow the polymerization process, while alkaline conditions promote faster setting. These dynamics are particularly relevant in the oral cavity, where variable pH due to saliva and microbial metabolism may subtly affect performance, requiring clinical awareness for optimal outcomes. In terms of cost-effectiveness, cyanoacrylate adhesives offer several advantages over traditional suturing. Although the initial material cost may be higher, these adhesives significantly reduce operative time and eliminate the need for suture removal appointments, which translates into lower overall healthcare costs. Additionally, their ease of application and reduced risk of postoperative complications may lead to shorter recovery times and higher patient satisfaction, further improving their cost–benefit profile in clinical practice. While cyanoacrylates offer numerous advantages, emerging concerns include the potential for biofilm formation and microbial resistance on treated wounds. Although current data suggest that cyanoacrylates inhibit early-stage bacterial colonization, there is a need for long-term studies to clarify their behaviour in polymicrobial environments typical of the oral cavity. This further research could provide valuable insights into the limitations of these adhesives and guide future clinical decision-making.

Equally important are patient-reported outcomes, which increasingly shape clinical decision-making. Preliminary studies report that patients experience less postoperative discomfort, reduced swelling, and shorter recovery periods when using cyanoacrylate adhesives instead of traditional sutures. These outcomes not only point to improved overall satisfaction but also instil optimism, especially in minimally invasive procedures.

Furthermore, pediatric patients and those unable to follow postoperative instructions (e.g., individuals with cognitive impairments) may not be ideal candidates, as the premature detachment of the adhesive layer could compromise wound integrity. Conversely, cyanoacrylate adhesives may be particularly well-suited for cardiopathic patients or those with significant cardiovascular disease, where achieving rapid and reliable hemostasis is critical. In such cases, the ability of cyanoacrylates to reduce intraoperative time and bleeding can offer substantial clinical advantages, minimizing surgical stress and enhancing overall procedural safety [14,16,17]. Notably, the specific formulation of Glubran II has been successfully adopted in various high-demand surgical fields, including cardiac surgery, pediatric cardiac surgery, and neurosurgery, where the time-sensitive control of bleeding and secure wound closure is essential. Its capacity to polymerize rapidly upon contact with tissue fluids reduces operative time. It provides an effective hemostatic seal, making it an ideal adjunct in complex and delicate procedures.

## 5. Conclusions

Applying cyanoacrylate adhesives, especially formulations like Glubran II, represents a paradigm shift in dentistry. Their ability to provide rapid hemostasis, facilitate tissue stabilization, and reduce procedural complications has positioned them as valuable tools in routine and complex dental surgeries. Studies by Mehta et al., Rezende et al., and Kulkarni et al. highlight their multifaceted benefits, ranging from faster healing in periodontal flaps to effective stabilization of bone grafts.

Despite these advancements, challenges such as limited osteointegration and potential local inflammatory responses indicate the need for further research. As noted in the works of Sagara et al. [25] and De Santis et al. [18], achieving long-term stability and integration with surrounding tissues remains a critical focus area. As explored by Choi et al. and others, emerging applications in areas like sinus membrane repair and implant stabilization point to a promising future for these adhesives in broader surgical contexts.

In conclusion, while cyanoacrylate adhesives have solidified their role in enhancing patient outcomes in dentistry, their full potential is yet to be realized. The ongoing innovation and clinical research, which are pivotal in overcoming current limitations, should inspire and motivate us. Cyanoacrylate adhesives, especially Glubran II, can become a gold standard in minimally invasive dental procedures. The research team aims to continue evaluating the long-term stability of the material and its behaviour in bone tissue and to explore new application techniques, contributing to the broader adoption and refinement of these adhesives in clinical practice.

## Figures and Tables

**Table 1:** The table compares three articles on the use of cyanoacrylates, indicating the procedures in which the product was applied and highlighting the key results obtained at the end of the study.

Study	Sample/Methods	Key Findings
Bhaskar et al. (1966) [7]	Applied on tongue lesions	Effective hemostasis; methyl variants showed higher abscess rates.
Ochstein (1969) [8]	Beagle dogs’ gingival wounds	Superior healing with butyl cyanoacrylate.
King et al. (1967) [9]	Tooth extraction in dogs	Durable hemostasis achieved with acrylic plugs.

**Table 2:** A comparison of two articles focused on the use of cyanoacrylates for bone regeneration and fixation, analyzing the results obtained in both studies.

Study	Methodology	Findings
Mehta et al. [15]	Mandibular fracture osteosynthesis	No adverse reactions over six months.
Salata et al. (2014) [17]	Rabbit mandibles with n-butyl adhesive	Reduced inflammation and improved mineralized tissue volume.

**Table 3:** The table compares the benefits of using cyanoacrylate-based adhesives in different types of dental procedures.

Application	Technique	Advantages
Socket Preservation	Bone graft with Glubran II	Stable graft fixation, reduced inflammation.
Post-Extraction Care	Adhesive seal on extraction sites	Decreased bleeding, minimized complications.

**Table 4:** A comparison of two articles focused on the use of cyanoacrylates for periodontal applications, comparing the results obtained with different techniques.

Study	Methodology	Key Results
Kulkarni et al. [20]	Flap procedures for periodontal pockets	Faster healing with adhesives versus sutures.
Ozcan et al. (2017) [23]	Free gingival graft healing	Enhanced palatal wound recovery.

**Table 5:** A comparison of two articles focused on the use of cyanoacrylates as a material for soft tissue stabilization, comparing the results obtained with different techniques.

Study	Applications	Outcomes
Giray et al. (1997) [4]	Root section stabilization	Low necrosis and enhanced aesthetics.
Rezende et al. [19]	Guided tissue regeneration	Favorable four-year outcomes.

**Table 6:** Description of clinical signs from clinical cases.

Clinical Case	Signs of Inflammation and Suppuration	No Inflammation/Suppuration
Case 1	Redness, Heat, Swelling	No
Case 2	Redness, Swelling	No
Case 3	Swelling, Pain	Yes
Case 4	Redness, Heat	No
Case 5	Heat, Pain, Swelling	Yes
Case 6	Redness, Swelling	Yes
Case 7	Redness, Heat, Pain, Loss of Function	No
Case 8	Swelling, Pain	Yes
Case 9	Heat, Redness, Swelling	No

## Data Availability

The original contributions presented in this study are included in the article. Further inquiries can be directed to the corresponding author.
